# A PILOT RCT TO EXAMINE THE IMPACT OF A THERAPY DOG INTERVENTION ON LONELINESS IN HOSPITALIZED OLDER ADULTS

**DOI:** 10.1093/geroni/igae098.4103

**Published:** 2024-12-31

**Authors:** Nancy Gee, Lisa Townsend, Erika Friedmann, Sandra Barker, Megan Mueller

**Affiliations:** Virginia Commonwealth University School of Medicine, Richmond, Virginia, United States; Virginia Commonwealth University, Richmond, Virginia, United States; University of Maryland Baltimore, Baltimore, Maryland, United States; Virginia Commonwealth University, Richmond, Virginia, United States; Tufts University, North Grafton, Massachusetts, United States

## Abstract

Loneliness is linked to significant health threats and is potentially more dangerous than obesity; it affects as many as 29% of noninstitutionalized older adults. Loneliness is exacerbated for those who require inpatient rehabilitation, are displaced from their social networks, spend little time receiving therapy, and are physically inactive and socially isolated. Evidence suggests that companion animals provide a number of health and wellbeing benefits and that interacting with a trained therapy dog may reduce loneliness. Older adult (59+ years) medical inpatients (N=42) were randomly assigned to receive one of three conditions: dog and handler interaction (AAI), handler-only Conversational Control (CC), or Usual Care (UC), for 20 minutes per day over three days. The UCLA Loneliness Scale and Short Form as well as an analog rating scale were used to assess loneliness. Linear Mixed Models with random intercepts were applied to examine differences in the changes from before to after the conditions. Changes in the UCLA-SF [t (200.356) = 1.851, p = 0.033] and the analog loneliness scale [t (194.407) = 2.651, p = 0.004], differed significantly between the AAI condition and the UC conditions but not between the CC and UC conditions (p =.175). Trajectories of changes in loneliness showed more improvement in the AAI than in the UC condition. These results indicate that AAI was effective for reducing loneliness in hospitalized older adults. Human handler only visits did not result in similar findings, indicating that there is something unique and beneficial about the presence of the dog.

